# Supplementary figures and images for: B cell–intrinsic requirement for WNK1 kinase in antibody responses in mice
Source: J Exp Med. 2023 Jan 20;220(3):e20211827. doi: 10.1084/jem.20211827 (PMC9872328; doi:10.1084/jem.20211827)

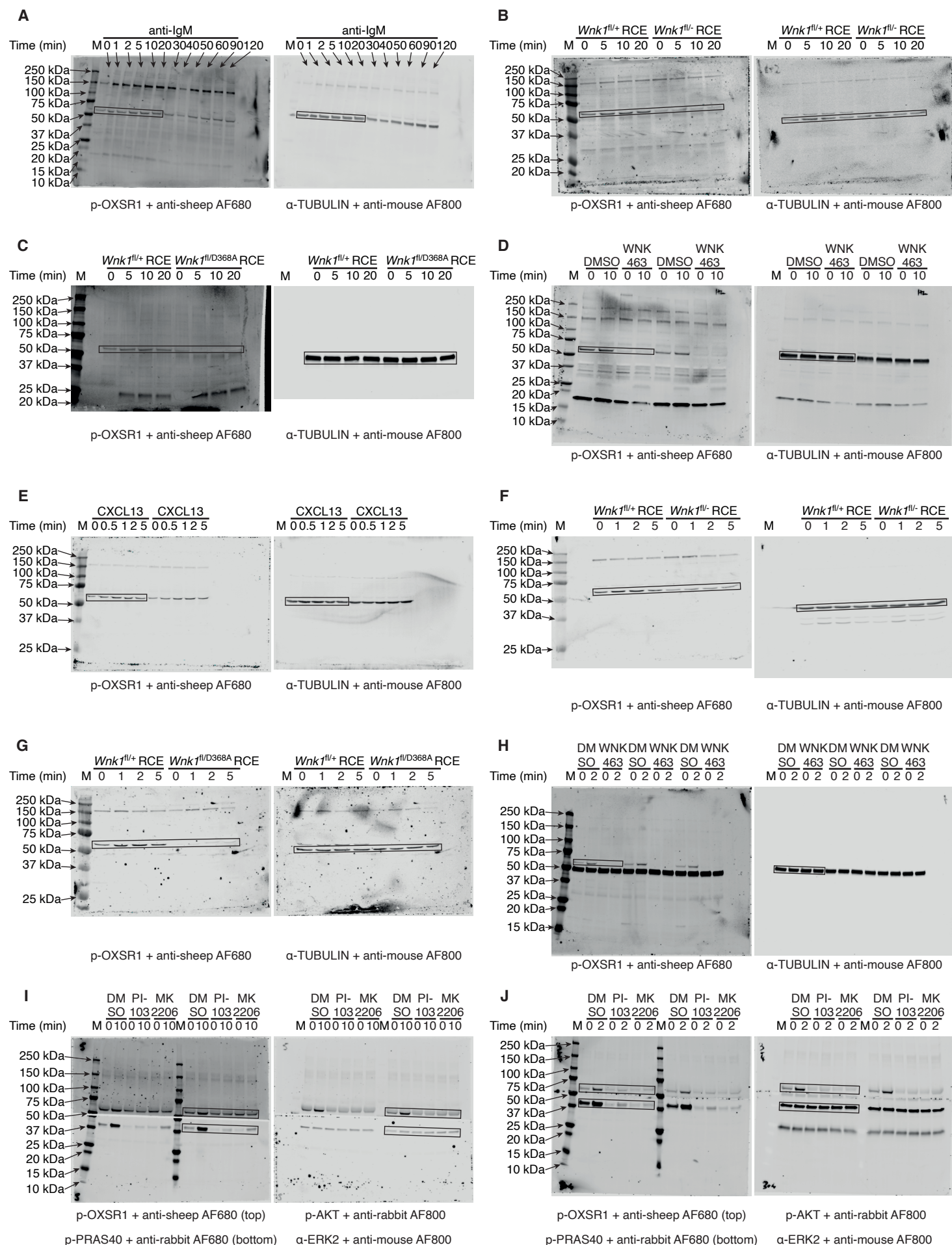

Supplement: SourceData F1 — contains original blots for Fig. 1. [file JEM_20211827_SourceDataF1.pdf]

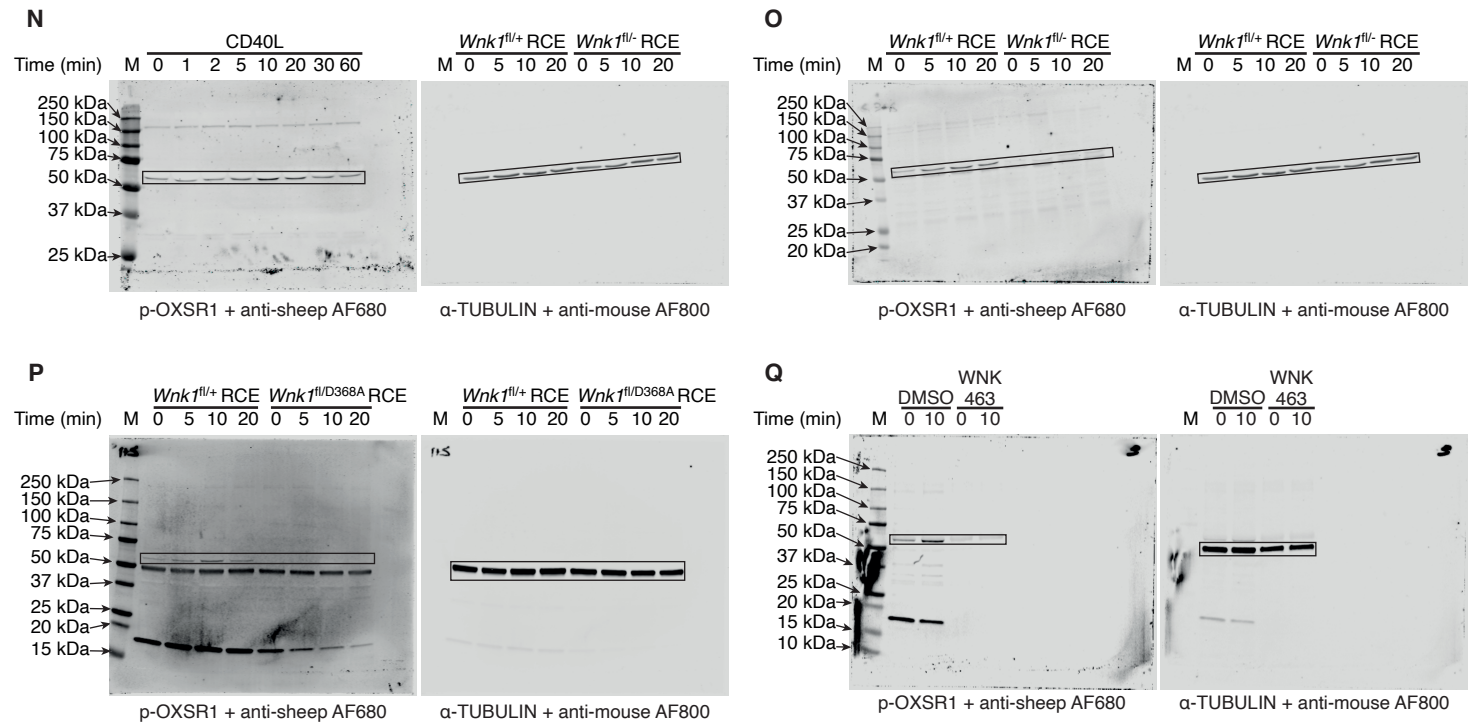

Supplement: SourceData F4 — contains original blots for Fig. 4. [file JEM_20211827_SourceDataF4.pdf]

**E**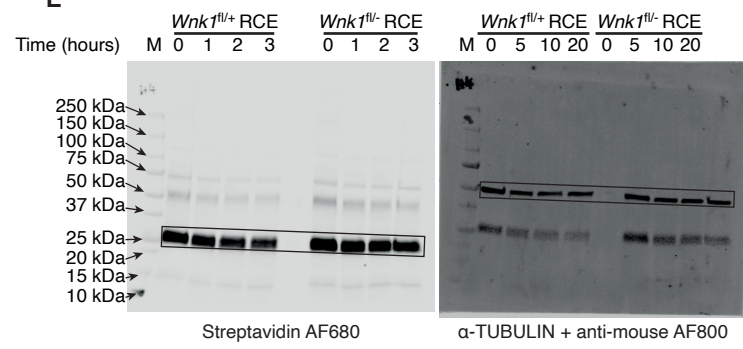

Supplement: SourceData F5 — contains original blots for Fig. 5. [file JEM_20211827_SourceDataF5.pdf]

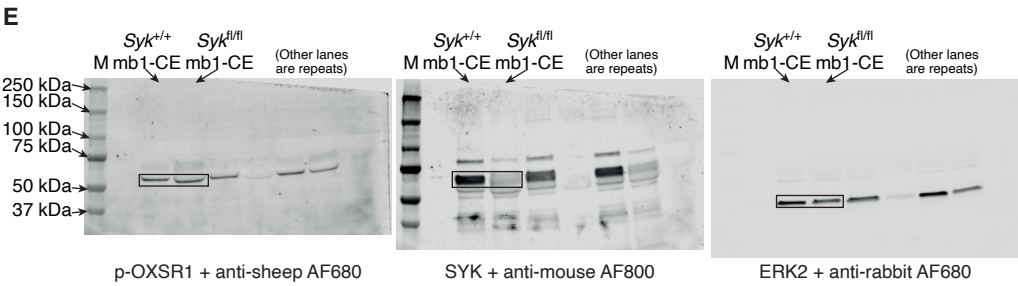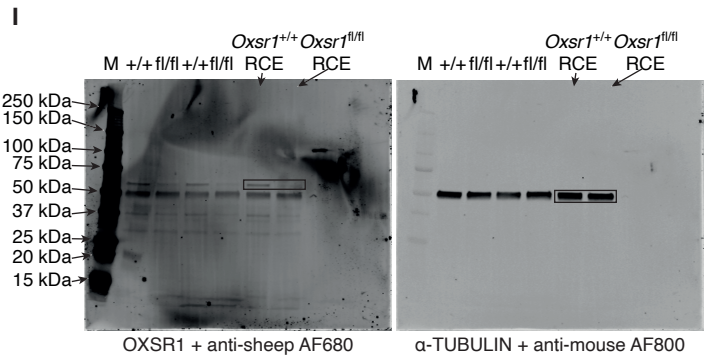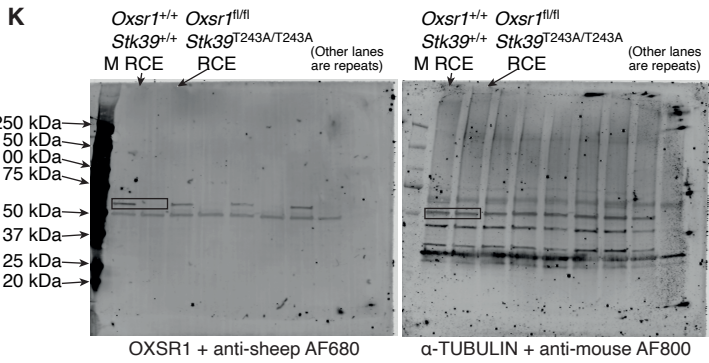

Supplement: SourceData FS2 — contains original blots for Fig. S2. [file JEM_20211827_SourceDataFS2.pdf]

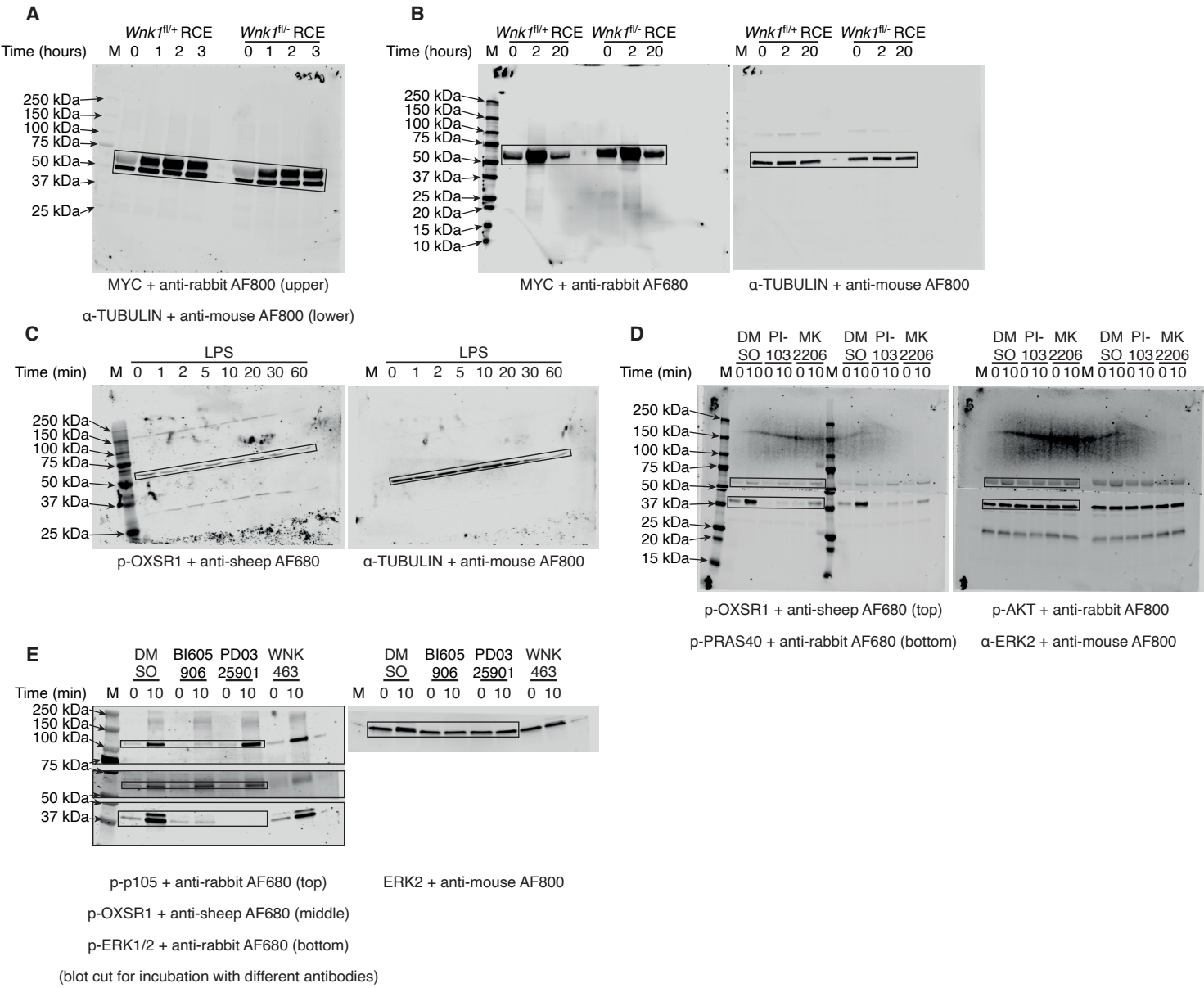

Supplement: SourceData FS4 — contains original blots for Fig. S4. [file JEM_20211827_SourceDataFS4.pdf]
